# Some Red Cross Activities

**Published:** 1912-02-17

**Authors:** 


### SOME RED CROSS ACTIVITIES.

### Voluntary Aid Detachments; and Instruction Manuals.

The activity and energy with which the British Red Cross Society threw itself into the arduous task of filling the gap in the organisation of the Medical Service of the Territorial Force are now matters of ancient history. This gap was one fatal to the Territorial Force, and was deliberately left unfilled in Lord Haldane's scheme because it was hoped that it would be filled by voluntary effort without cost to the taxpayer. Critics of the scheme dwelt upon the unsound and dangerous principle of thus leaving important matters to chance, and especially of leaving them unfinanced. It was contended that this flaw would prove fatal to the working of the force, and that voluntary effort would fail to compensate adequately for the weakness of the Territorial scheme in this respect.

The old-established St. John Ambulance Association saw the serious financial risk which it would incur by guaranteeing to raise the material and personnel for this voluntary unpaid work, and declined the responsibility. The then newly reconstituted Red Cross Society, having less to risk, rushed into the breach, and has ever since done its best to fill it worthily.

### Women and the War Office.

As a matter of fact, the response of the public, especially of the leisured classes, in personal service and enthusiasm has surprised even those who thought most of British patriotism. If keenness and hard work were all, the success of the movement is long ago assured. Nor must we omit to mention that the chief honours in this respect rest with the women of Britain, who have done wonders. The War Office, as usual, having allowed an organisation, which it now finds to be a bother, to come into existence, pursues its old policy of alternate pinpricks and neglect in the hope of saving itself the trouble of supervision. It was certainly difficult to see why people should be such fools as to work for their country and also pay for the honour of doing it; but still they did it in large numbers, and now they are asking the War Office for a little straw to make bricks with. This reasonable request will no doubt be refused as long as possible; but The Hospital has already pointed out (January 20, p. 410) that justice endorses the demand, and in time it is certain to be conceded if only the workers will press it.

### Voluntary Aid Efficiency.

Meanwhile a large number of *Voluntary Aid Detachments* have come into existence all over the kingdom, and a corresponding number of commandants and medical men are responsible for their efficiency. The functions of these Voluntary Aid Detachments are partly quite novel; no precedent exists for their work, and text-books are needed for their instruction. As far as first aid and elementary nursing are concerned, the excellent manuals of the St. John Ambulance Association supply the want. That on First Aid by Mr. James Cantlie, of which more than half a million copies have been sold, is an old friend, and contains everything necessary. In some respects it even exceeds requirements; and a briefer manual has just been issued by Dr. D. M. Macdonald (Gill and Sons, Limited, 1s. net). This follows on approved lines, and contains hints for lecturers as well as for pupils. It has been specially designed to meet Red Cross requirements and will doubtless prove valuable to those who find the St. John text-book unduly voluminous or exacting. Official Red Cross text-books on First Aid and Home Nursing are also published.

### Mr. Cantlie's Guide to Training.

Another manual just issued is perhaps of greater interest at the moment, since it breaks new ground altogether. This is the British Red Cross Society's Training Manual, by Mr. Cantlie, published by Cassell's at the price of one shilling. This extremely interesting publication is intended to guide those who have already acquired a knowledge of first aid and nursing.

It is primarily a guide to the direction in which training in improvised methods of assistance to the sick and wounded is to be carried out. It is the third and concluding volume of Mr. Cantlie's admirable series. Without entering into detailed criticism, it is possible to affirm that when the 31,000 members of the 1,063 Red Cross Voluntary Aid Detachments already formed have mastered this manual and those to which it is the sequel, the gap in the Territorial Force Medical Service will be filled in so far as the energy and patriotism of the members can fill it.

The duties of the Voluntary Aid Detachments are, broadly, to manage the transference of the wounded from the Field Ambulances on the battle-field to the hospitals in the towns; and to improvise the whole of the transport, equipment, stretchers, tents, food, dressings, and a thousand other necessities which such transfer will entail. Mr. Cantlie's book explains how this arduous task can be attempted. The practical note is thoroughly well-sustained all through the manual, and the advice given is sound and dependable. On one small point a popular misconception is adopted; its very minuteness shows how trustworthy Mr. Cantlie is about essentials.

The point is that it is the great trochanter of the thigh, not the margin of the ilium or "hip-bone," which gets bruised and sore when men have to sleep out on hard ground. After digging a depression, therefore, the old campaigner fits the thighbone into it, not the hip, as most people believe. One other detail seems to be unusual; in the instructions for pricking a blister, it is recommended to pass a threaded needle into the sound skin close to the edge of the blister and out at a corresponding point on the other side. This drastic treatment must be unnecessarily painful, though we admit we have not put it to the test of performance.

